# Does Pre-operative Biliary Drainage Influence Long-Term Survival in Patients With Obstructive Jaundice With Resectable Pancreatic Head Cancer?

**DOI:** 10.3389/fonc.2020.575316

**Published:** 2020-09-16

**Authors:** Ziyun Shen, Jun Zhang, Haoda Chen, Weishen Wang, Wei Xu, Xiongxiong Lu, Yiran Zhou, Shiwei Zhao, Zhiwei Xu, Xiaxing Deng, Jiancheng Wang, Yuanchi Weng, Baiyong Shen

**Affiliations:** ^1^Department of General Surgery, Pancreatic Disease Center, Ruijin Hospital, Shanghai Jiao Tong University School of Medicine, Shanghai, China; ^2^Institute of Translational Medicine, Shanghai Jiao Tong University, Shanghai, China; ^3^Research Institute of Pancreatic Disease, Shanghai Jiao Tong University School of Medicine, Shanghai, China; ^4^State Key Laboratory of Oncogenes and Related Genes, Shanghai, China

**Keywords:** obstructive jaundice, pre-operative biliary drainage, pancreaticoduodenectomy, pancreatic cancer, long-term survival

## Abstract

**Objectives:** Whether pre-operative biliary drainage (PBD) affects long-term survival of patients with obstructive jaundice with pancreatic ductal adenocarcinoma (PDAC) who underwent pancreaticoduodenectomy is still controversial. Most of the previous research did not include the important total serum bilirubin (TB) level before intervention as well as before surgery. The aim of this study is to evaluate the impact of PBD on long-term survival after considering the TB level.

**Methods:** Data were collected retrospectively from patients with obstructive jaundice who underwent resection of pancreatic head cancer in a high-volume center. X-Tile software and Kaplan-Meier survival analysis were applied to determine the optimal cut-off levels for TB and age based on the minimal probability (*P*)-value and the largest χ2-value. Multivariate Cox regression analyses were performed after univariate analysis to assess independent prognostic factors for overall survival (OS) and disease-free survival (DFS).

**Results:** Of 426 patients with obstructive jaundice who underwent pancreaticoduodenectomy for resectable pancreatic head cancer during a 7 year period, 242 (56.8%) received PBD and 184 (43.2%) underwent surgery directly. The OS of patients who received PBD was significantly worse than that of patients who did not receive PBD by univariate analysis (median of 16.6 vs. 22.2 months, *P* = 0.048). After including liver function parameters in the multivariate Cox regression, we found that the use of PBD was not associated with OS or DFS, while TB before intervention >150 μmol/L was an independent adverse prognostic factor for both OS [hazard ratio (HR), 1.42; 95% CI, 1.05–1.91] and DFS (HR, 1.38; 95% CI, 1.08–1.77).

**Conclusions:** In patients with obstructive jaundice with resectable pancreatic head cancer, undergoing PBD before pancreaticoduodenectomy did not impair or benefit survival rates compared with surgery alone. However, TB before intervention >150 μmol/L predicted an unfavorable prognosis, irrespective of the PBD procedure.

## Introduction

The prevalence of pancreatic cancer has increased in the last decade to become the fourth leading cause of cancer death in the United States (1). As its incidence continues to increase, with a 5 year survival rate of <10%, pancreatic cancer remains a fatal disease for most patients. Although pancreaticoduodenectomy is one of the most complex surgeries and is associated with considerable morbidity and mortality, it is still the only potential therapy to treat patients with resectable pancreatic ductal adenocarcinoma (PDAC).

The most common symptom in patients with pancreatic head cancer is obstructive jaundice. Accordingly, pre-operative biliary drainage (PBD) has been developed to relieve biliary obstruction and aims to potentially reduce post-operative complications by improving liver function pre-operatively. Although routine PBD could not reduce morbidity compared to surgery alone according to previous studies, a considerable proportion of patients could benefit from PBD under strict clinical indications (2–5).

The influence of PBD on short-term outcomes of patients with obstructive jaundice has received increased attention in recent years. In contrast, there has been little agreement about whether PBD has an adverse effect on the long-term survival of patients with obstructive jaundice who underwent resection of pancreatic head cancer. Several studies have reported that the use of PBD had an unfavorable impact on the long-term survival of patients with resected PDAC, while other studies have failed to find a connection between PBD and long-term survival (6–10). However, several studies have found that obstructive jaundice is a negative risk factor in patients with pancreatic head cancer (11–13). Thus, previous studies of the impact of PBD on long-term survival in all patients have suffered from shortcomings in the methods used to select cases. Furthermore, the duration of PBD, which was rarely included in the previous research, may also influence the long-term outcome in patients with obstructive jaundice based on the recent studies (14, 15).

The purpose of this study was to explore the relationship between the severity of obstructive jaundice, PBD, duration of PBD, and long-term survival in patients with obstructive jaundice with resectable pancreatic head cancer.

## Methods

### Study Design

Clinical data were collected retrospectively in the Pancreatic Surgery Department of Ruijin Hospital, Shanghai Jiao Tong University School of Medicine for consecutive patients who underwent pancreaticoduodenectomy for PDAC between January 2012 and December 2018. Included patients were 18–85 years of age and presented with obstructive jaundice, which was defined as a total serum bilirubin (TB) level exceeding 34.2 μmol/L (2 mg/dL) before intervention (16). All patients with locally advanced pancreatic cancer or metastatic disease by computed tomography or during surgery were excluded. Patients who received neoadjuvant chemotherapy were also excluded.

### PBD Procedure

PBD was performed by using endoscopic retrograde cholangiopancreatography (ERCP) or percutaneous transhepatic cholangiodrainage (PTCD) in patients with acute cholangitis, severe malnutrition, contraindications to surgery or in whom surgical resection was significantly delayed. The PBD methods used on patients were determined by surgeons and interventional radiologists. Additionally, in case of biliary infection related to PBD, patients received antibiotic therapy that was monitored by surgeons. The intervals between PBD and surgery were recorded using the medical records of patients.

### Data Collection

The pre-operative data available closest to the time of surgery were collected and used for analysis. For patients who underwent PBD, TB and serum albumin were also collected before PBD when available. What needs illustration is that the TB before intervention was defined as the TB before PBD in patients who underwent PBD as well as the TB before surgery in patients who did not undergo PBD. Neutrophil to lymphocyte ratio (NLR) was defined as the absolute neutrophil count divided by the absolute lymphocyte count.

The intraoperative information included surgical method [i.e., open pancreaticoduodenectomy (OPD) or minimally invasive pancreaticoduodenectomy (MIPD)], venous resection, and resection margins.

Post-operative pathologic stage was classified based on the 8th edition of the American Joint Committee on Cancer (AJCC) TNM staging system. The grade of tumor differentiation was also collected. Post-operative information also included post-operative complications and adjuvant chemotherapy.

### Follow-Up

All the patients with resected pancreatic cancer in our department were followed up at regular intervals. A telephone follow-up call was made every 3 months with informed consent of the patient, and the follow-up ended with the patient's death. Patients were evaluated for recurrence based on computed tomography (CT), magnetic resonance imaging (MRI), or positron emission tomography-computed tomography (PET/CT). Survival end points included overall survival (OS) and disease-free survival (DFS). OS of the patients was defined as the time elapsed from pancreaticoduodenectomy to death from any cause. DFS was defined as the surgery to recurrence or death from any cause, whichever occurred first. Survival data were censored at the date of last follow-up.

### Statistical Analysis

Data were expressed as medians (ranges) when they showed normal distribution or expressed as medians (interquartile ranges) when they did not. In order to determine the optimal cut-off point of TB level for maximum OS difference, X-tile software version 3.6.1 was used (17). The same method was used to determine the optimal cut-off point for age. The transformation of the albumin level into categories was made based on the lower normal reference value (35 g/L).

Categorical data were compared using Pearson's chi-square test or Fisher's exact test where appropriate. Paired *t*-tests were also used to analyse paired data. Survival curves were constructed using the Kaplan-Meier method with the log-rank test. Only factors found to be significant in the univariate analysis were entered into the multivariate Cox regression analysis. The Harrell concordance index (C-index) was calculated to measure the performance of the Cox model.

All statistical analyses were performed using X-tile software version 3.6.1 and R version 3.6.3. All tests were two-tailed, and *P* < 0.05 was considered statistically significant.

## Results

### Patient Characteristics

During the 7 year study period, a total of 426 patients with obstructive jaundice due to PDAC were included. Baseline clinicopathological characteristics for the study population are listed in Table 1. There were 287 males (67.4%), and the mean age was 63.4 ± 9.6 years. Among them, 242 (56.8%) patients received PBD, 107 (25.1%) via ERCP, and 135 (31.7%) via PTCD, respectively. The median elapsed time from PBD to surgery was 2 weeks. The median TB before surgery was significantly lower than the median TB before intervention in all cohorts (132 μmol/L vs. 216 μmol/L, *P* < 0.001). Meanwhile, the albumin before surgery was significantly higher than the albumin before intervention (*P* < 0.001).

**Table 1:** Baseline clinicopathological characteristics.

**Variables**	**Number**
Total number	426
Sex (%)	
Male	287 (67.4%)
Female	139 (32.6%)
Mean age (SD), years	63.4 (9.6)
TB before intervention [median (IQR)], μmol/L	216 (138, 297)
Albumin before intervention [median (IQR)], g/L	35 (32, 38)
PBD procedure (%)	
No PBD	184 (43.2%)
ERCP	107 (25.1%)
PTCD	135 (31.7%)
Elapsed time from PBD to surgery [median (IQR)], weeks	2 (1, 3)
TB before surgery [median (IQR)], μmol/L	132 (84, 199)
Albumin before surgery [median (IQR)], g/L	36 (33, 39)
NLR [median (IQR)]	3.2 (2.3, 4.4)
Surgical method (%)	
OPD	362 (85.0%)
MIPD	64 (15.0%)
Venous resection (%)	
No	376 (88.3%)
Yes	50 (11.7%)
T stage (%)	
T1	49 (11.5%)
T2	254 (59.6%)
T3	60 (14.1%)
T4	52 (12.2%)
Missing	11 (2.6%)
N stage (%)	
N0	203 (47.7%)
N1	162 (38.0%)
N2	50 (11.7%)
Missing	11 (2.6%)
Differentiation (%)	
Well	123 (28.9%)
Moderate	203 (47.7%)
Poor	85 (20.0%)
Missing	15 (3.5%)
Resection margins (%)	
R0	332 (77.9%)
R1	72 (16.9%)
R2	22 (5.2%)
Post-operative complication (%)	
No	238 (55.9%)
Yes	188 (44.1%)
Adjuvant chemotherapy (%)	
No	198 (46.5%)
Yes	228 (53.5%)

*ERCP, endoscopic retrograde cholangiopancreatography; IQR, interquartile range; MIPD, minimally invasive pancreaticoduodenectomy; NLR, neutrophil-lymphocyte ratio; OPD, open pancreaticoduodenectomy; PBD, pre-operative biliary drainage; PTCD, percutaneous transhepatic cholangiodrainage; SD, standard deviation; TB, total serum bilirubin*.

### Survival Information and Optimal Cut-Off Point

At a median follow-up time of 16.7 months (range, 0.2–80.8 months), the median OS time, and the median DFS time were 18.5 and 12.3 months, respectively. At the last follow-up, 112 (26.3%) patients were still alive and 314 (73.7%) patients had died. Five patients were lost from the study population.

**Figure 1:**
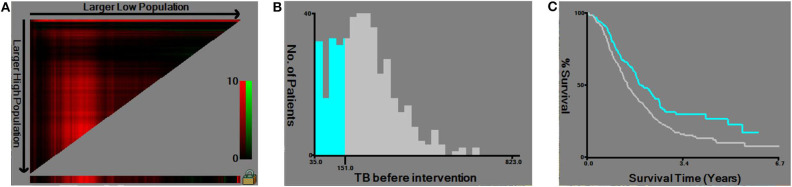
Utilizing X-tile analysis to determine the optimal cut-off level of the total serum bilirubin (TB) before intervention. **(A)** The graph shows that the optimal cut-off point has been determined by X-tile software. **(B)** Histogram and **(C)** Kaplan-Meier analysis were conducted using the optimal cut-off value.

**Figure 2:**
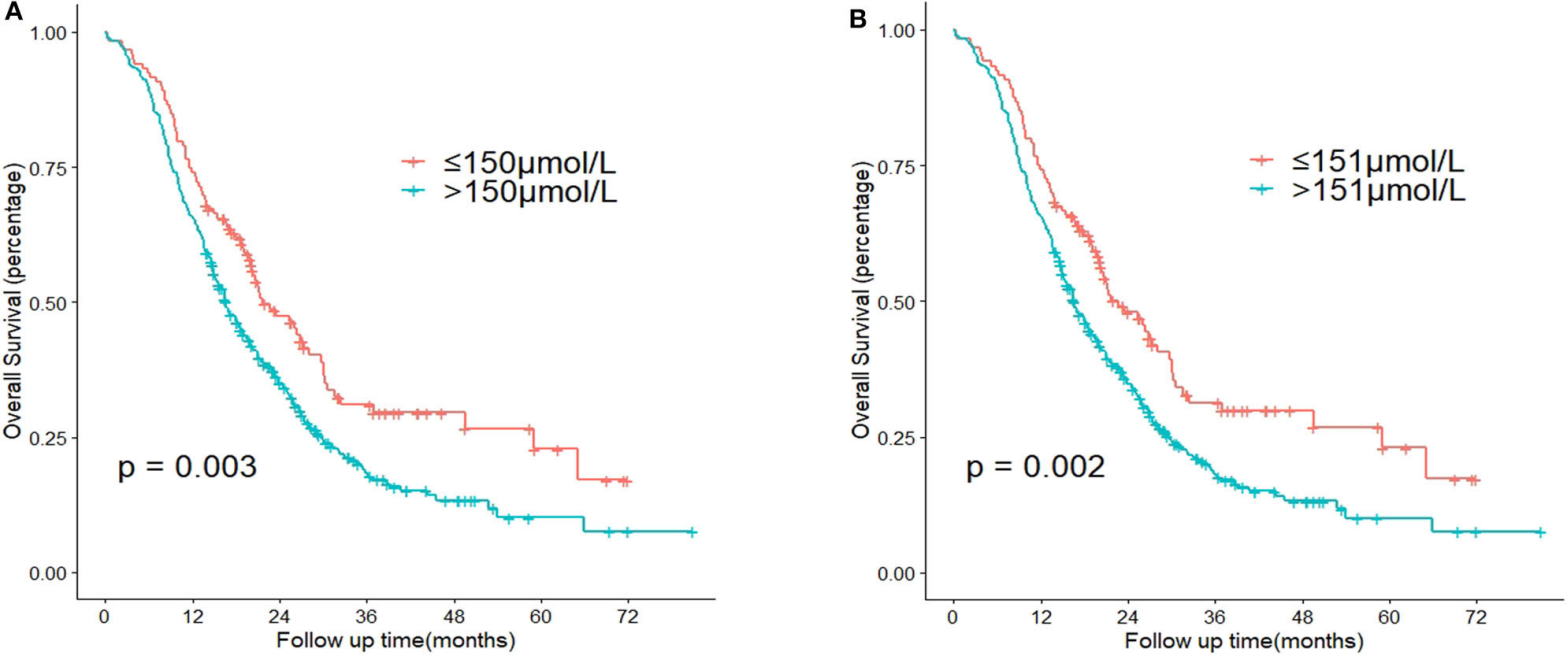
Comparison of *P*-value according to the cut-off point of total serum bilirubin (TB) before intervention using Kaplan-Meier analysis. **(A)** Cut-off point = 150 μmol/L. **(B)** Cut-off point = 151 μmol/L.

According to the X-tile software, the optimal cut-off point of TB before intervention was 151 μmol/L (≤ 151 μmol/L vs. >151 μmol/L, *P* = 0.002) (Figure 1). Note the slight difference of cut-off point between 150 μmol/L and 151 μmol/L (*P* = 0.003 and *P* = 0.002, respectively) (Figure 2). With regard to the feasibility for clinical application, we set 150 μmol/L as the best cut-off point for TB before intervention. The optimal cut-off point of age was 65 years (≤ 65 vs. >65 years, *P* < 0.001) (Supplementary Figure 1). The optimal cut-off point of TB before surgery was 136 μmol/L (≤ 136 μmol/L vs. >136 μmol/L, *P* = 0.060) (Supplementary Figure 2).

Patient characteristics were compared between the two groups based on their PBD status after X-tile analysis (Table 2). Patients who received PBD had higher levels of TB before intervention (*P* < 0.001). Furthermore, patients who received PBD had lower levels of albumin before intervention (*P* < 0.001), meaning these patients may have worse liver function. After PBD, patients had lower levels of TB before surgery compared to patients who did not have the PBD procedure (*P* = 0.001).

**Table 2:** Comparison of baseline clinicopathological characteristics based on PBD status.

**Variables**	**No PBD**	**PBD**	***P***
Total number	184	242	
Sex (%)			
Male	121 (65.8)	166 (68.6)	0.607
Female	63 (34.2)	76 (31.4)	
Age (%), years			
≤ 65	109 (59.2)	133 (55.0)	0.433
>65	75 (40.8)	109 (45.0)	
TB before intervention (%), μmol/L			
≤ 150	89 (48.4)	30 (12.4)	<0.001
>150	95 (51.6)	212 (87.6)	
Albumin before intervention (%), g/L			
<35	68 (37.0)	132 (54.5)	<0.001
≥35	116 (63.0)	110 (45.5)	
Elapsed time from PBD to surgery (%), weeks			
<4	NA	202 (83.5)	
≥4	NA	40 (16.5)	
TB before surgery (%), μmol/L			
≤ 150	89 (48.4)	158 (65.3)	0.001
>150	95 (51.6)	84 (34.7)	
Albumin before surgery (%), g/L			
<35	67 (36.4)	93 (38.4)	0.745
≥35	117 (63.6)	149 (61.6)	
NLR (%)			
≤ 2	31 (16.8)	40 (16.5)	1.000
>2	153 (83.2)	202 (83.5)	
Surgery method (%)			
OPD	160 (87.0)	202 (83.5)	0.390
MIPD	24 (13.0)	40 (16.5)	
Venous resection (%)			
No	164 (89.1)	212 (87.6)	0.739
Yes	20 (10.9)	30 (12.4)	
T stage (%)			
T1–3	159 (88.3)	204 (86.8)	0.642
T4	21 (11.7)	31 (13.2)	
N stage (%)			
N0	92 (51.1)	111 (47.2)	0.434
N1/N2	88 (48.9)	124 (52.8)	
Differentiation (%)			
Good	60 (33.7)	63 (27.0)	0.143
Moderate–Poor	118 (66.3)	170 (73.0)	
Resection margins (%)			
Negative	142 (77.2)	190 (78.5)	0.832
Positive (R1/R2)	42 (22.8)	52 (21.5)	
Post-operative complication (%)			
No	108 (58.7)	130 (53.7)	0.354
Yes	76 (41.3)	112 (46.3)	
Adjuvant chemotherapy (%)			
No	79 (42.9)	119 (49.2)	0.238
Yes	105 (57.1)	123 (50.8)	

*MIPD, minimally invasive pancreaticoduodenectomy; NLR, neutrophil-lymphocyte ratio; OPD, open pancreaticoduodenectomy; PBD, pre-operative biliary drainage; TB, total serum bilirubin*.

### Risk Factors for OS and DFS

**Figure 3:**
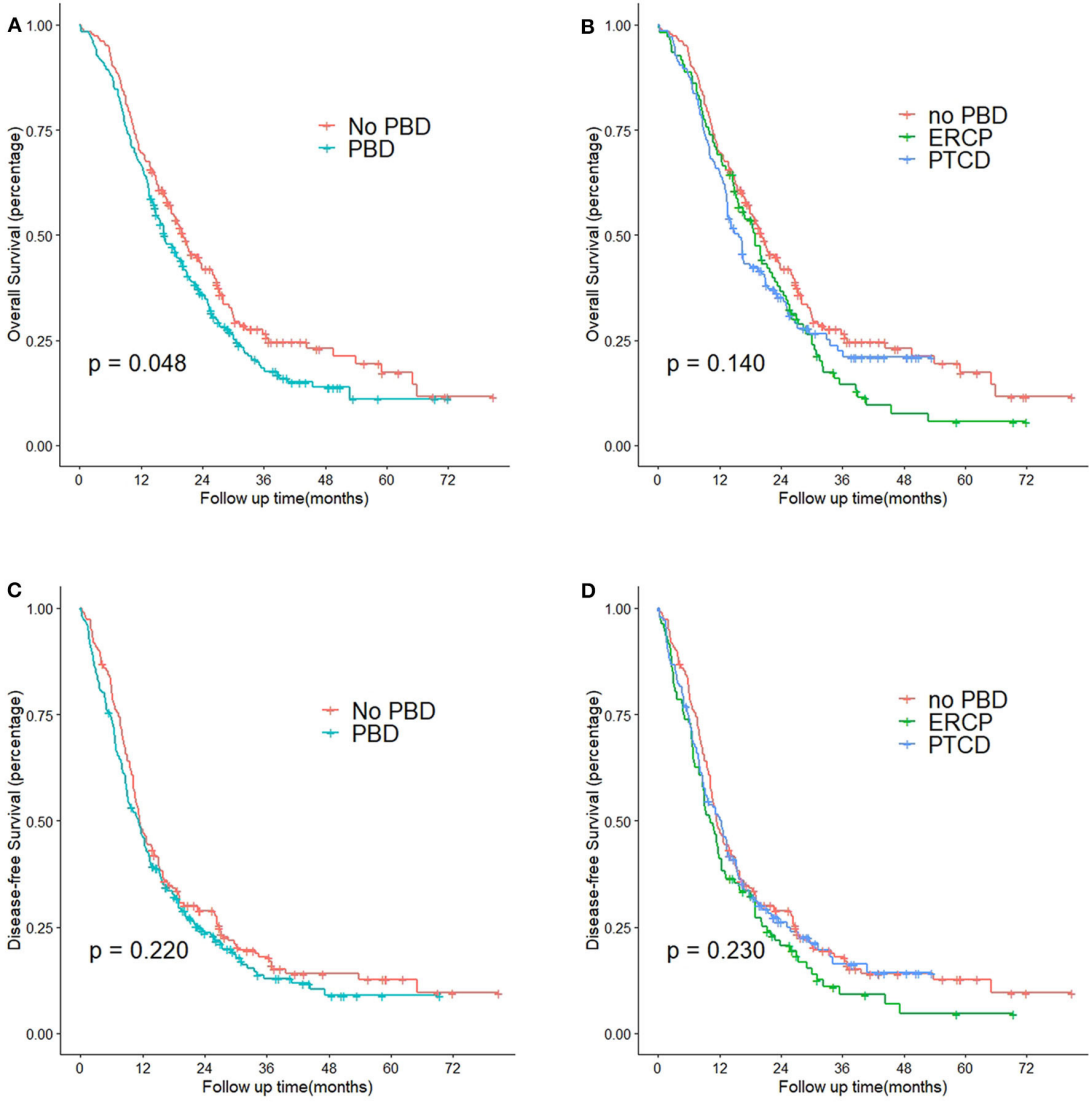
Kaplan-Meier curves of patients with obstructive jaundice with resectable pancreatic head cancer who did or did not undergo PBD. **(A)** OS curves of patients who did or did not undergo PBD (*P* = 0.048). **(B)** OS curves of patients who did not undergo PBD, patients who underwent ERCP, and patients who underwent PTCD (*P* = 0.140). **(C)** DFS curves of patients who did or did not undergo PBD (*P* = 0.220). **(D)** DFS curves of patients who did not undergo PBD, patients who underwent ERCP, and patients who underwent PTCD (*P* = 0.230). PBD, pre-operative biliary drainage; ERCP, endoscopic retrograde cholangiopancreatography; PTCD, percutaneous transhepatic cholangiodrainage.

Figure 3 shows the Kaplan-Meier curves for OS and DFS among the patients with PDAC stratified by PBD status and type of PBD. Patients who received the PBD procedure (green) had significantly poorer OS than patients undergoing pancreaticoduodenectomy directly (red) (median of 16.6 vs. 22.2 months, *P* = 0.048). None of the other comparisons were statistically significant.

The results of the uni- and multivariate analyses of prognostic factors for OS in patients with obstructive jaundice are shown in Table 3. Displayed are the hazard ratios (HRs) and their 95% confidence intervals (CIs). In the univariate analysis, age, PBD status, TB before intervention, albumin before intervention, NLR, venous resection, T stage, N stage, differentiation, resection margins, and adjuvant chemotherapy were significantly associated with prognosis. To control for potential confounding factors in the patients with obstructive jaundice while investigating the potential relationship between liver function parameters and patient prognosis, we compared two Cox regression models: model 1 did not contain any liver function parameters, and model 2 contained liver function parameters that were significant in the univariate analysis. Therefore, in model 1, the multivariate analysis showed that age >65 years (*P* = 0.003), PBD (*P* = 0.026), NLR >2 (*P* = 0.011), venous resection (*P* = 0.010), positive lymph node metastasis (*P* = 0.024), poor or moderate differentiation (*P* = 0.049), positive surgical margin status (*P* = 0.002), and no completion of adjuvant chemotherapy (*P* = 0.001) remained independently associated with worse OS. When liver function parameters were enrolled, model 2 showed that PBD did not affect OS (*P* = 0.428). Meanwhile, both TB before intervention >150 μmol/L and albumin before intervention <35 g/L were independent risk factors for OS (*P* = 0.022 and *P* = 0.022, respectively). We also used the C-index as the evaluation criteria. The C-index for model 2 was 0.681, which was higher compared to model 1 (C-index = 0.676).

**Table 3:** Risk factors of overall survival: univariate and multivariate analyses.

	**Univariate**		**Multivariate (Model 1)**		**Multivariate (Model 2)**	
**Variable**	**HR (95% CI)**	***P***	**HR (95% CI)**	***P***	**HR (95% CI)**	***P***
Sex, female vs. male	1.04 (0.82–1.32)	0.758				
Age, years, >65 vs. ≤ 65	1.63 (1.30–2.03)	<0.001	1.46 (1.14–1.86)	0.003	1.45 (1.14–1.86)	0.003
PBD status, yes vs. no	1.25 (1.00–1.57)	0.049	1.31 (1.03–1.65)	0.026	1.11 (0.86–1.44)	0.428
TB before intervention, μmol/L, >150 vs. ≤ 150	1.48 (1.14–1.92)	0.003	NA		1.42 (1.05–1.91)	0.022
Albumin before intervention, g/L, ≥35 vs. <35	0.75 (0.60–0.93)	0.010	NA		0.76 (0.60–0.96)	0.022
Time between PBD and surgery, weeks, ≥4 vs. <4	1.09 (0.74–1.60)	0.668				
TB before surgery, μmol/L, >150 vs. ≤ 150	1.16 (0.92–1.44)	0.205				
Albumin before surgery, g/L, ≥35 vs. <35	0.84 (0.67–1.06)	0.138				
NLR, >2 vs. ≤ 2	1.63 (1.19–2.23)	0.003	1.52 (1.10–2.11)	0.011	1.54 (1.11–2.14)	0.009
Surgical method, MIPD vs. OPD	0.82 (0.59–1.14)	0.235				
Venous resection, yes vs. no	1.49 (1.08–2.07)	0.016	1.57 (1.11–2.21)	0.010	1.71 (1.21–2.43)	0.003
T stage, T4 vs. T1–3	1.71 (1.24–2.35)	0.001	1.33 (0.95–1.86)	0.093	1.37 (0.98–1.92)	0.067
N stage, N1/N2 vs. N0	1.39 (1.11–1.74)	0.004	1.31 (1.04–1.66)	0.024	1.25 (0.99–1.59)	0.063
Differentiation, poor/moderate vs. good	1.41 (1.10–1.81)	0.007	1.29 (1.00–1.67)	0.049	1.26 (0.98–1.63)	0.071
Resection margins, R1/R2 vs. R0	1.51 (1.17–1.96)	0.002	1.54 (1.17–2.02)	0.002	1.60 (1.22–2.11)	0.001
Post-operative complication, yes vs. no	1.13 (0.90–1.41)	0.287				
Adjuvant chemotherapy, yes vs. no	0.59 (0.47–0.73)	<0.001	0.66 (0.52–0.84)	0.001	0.69 (0.54–0.87)	0.002

*MIPD, minimally invasive pancreaticoduodenectomy; NLR, neutrophil-lymphocyte ratio; OPD, open pancreaticoduodenectomy; PBD, pre-operative biliary drainage; TB, total serum bilirubin*.

Table 4 shows the results of the uni- and multivariate analyses of risk factors for DFS in patients with obstructive jaundice. TB before intervention was an independent risk factor, but PBD status was not an independent risk factor. Two models were constructed in the same way as noted above. In model 1, the multivariate analysis showed that age >65 years (*P* = 0.027), NLR >2 (*P* = 0.006), venous resection (*P* = 0.010), positive lymph node metastasis (*P* = 0.016), poor or moderate differentiation (*P* = 0.023), and positive surgical margin status (*P* = 0.020) affected DFS. We then analyzed the DFS when TB before intervention was added to the model. In model 2, TB before intervention >150 μmol/L was identified as an independent adverse prognostic factor, while the other parameters were still significant, as in model 1. The C-index for model 2 was 0.631, which was higher compared to model 1 (C-index = 0.622).

**Table 4:** Risk factors of disease-free survival: univariate and multivariate analyses.

	**Univariate**		**Multivariate (Model 1)**		**Multivariate (Model 2)**	
**Variable**	**HR (95% CI)**	***P***	**HR (95% CI)**	***P***	**HR (95% CI)**	***P***
Sex, female vs. male	1.07 (0.85–1.34)	0.557				
Age, years, >65 vs. ≤ 65	1.27 (1.02–1.57)	0.029	1.29 (1.03–1.61)	0.027	1.28 (1.02–1.60)	0.031
PBD status, yes vs. no	1.14 (0.92–1.41)	0.225				
TB before intervention, μmol/L, >150 vs. ≤ 150	1.32 (1.04–1.67)	0.024	NA		1.38 (1.08–1.77)	0.010
Albumin before intervention, g/L, ≥35 vs. <35	0.86 (0.69–1.06)	0.149				
Time between PBD and surgery, weeks, ≥4 vs. <4	0.97 (0.66–1.43)	0.875				
TB before surgery, μmol/L, >150 vs. ≤ 150	1.12 (0.91–1.39)	0.283				
Albumin before surgery, g/L, ≥35 vs. <35	0.93 (0.75–1.15)	0.490				
NLR, >2 vs. ≤ 2	1.60 (1.19–2.17)	0.002	1.54 (1.13–2.10)	0.006	1.55 (1.14–2.12)	0.005
Surgical method, MIPD vs. OPD	0.88 (0.65–1.19)	0.397				
Venous resection, yes vs. no	1.53 (1.12–2.10)	0.008	1.54 (1.11–2.14)	0.010	1.56 (1.12–2.17)	0.008
T stage, T4 vs. T1–3	1.59 (1.16–2.16)	0.003	1.23 (0.89–1.71)	0.207	1.26 (0.91–1.75)	0.162
N stage, N1/N2 vs. N0	1.43 (1.15–1.78)	0.001	1.32 (1.05–1.65)	0.016	1.30 (1.04–1.63)	0.022
Differentiation, poor/moderate vs. good	1.37 (1.08–1.74)	0.009	1.32 (1.04–1.68)	0.023	1.31 (1.03–1.66)	0.030
Resection margins, R1/R2 vs. R0	1.43 (1.12–1.83)	0.005	1.37 (1.05–1.78)	0.020	1.43 (1.10–1.87)	0.008
Post-operative complication, yes vs. no	1.08 (0.88–1.34)	0.467				
Adjuvant chemotherapy, yes vs. no	0.87 (0.70–1.07)	0.181				

*MIPD, minimally invasive pancreaticoduodenectomy; NLR, neutrophil-lymphocyte ratio; OPD, open pancreaticoduodenectomy; PBD, pre-operative biliary drainage; TB, total serum bilirubin*.

## Discussion

The initial objective was to assess the impact of PBD on long-term survival in patients with obstructive jaundice. With respect to the first research question, it was found that PBD may have a negative impact on prognosis after pancreaticoduodenectomy in model 1 (Table 3), as mentioned above. Contrary to expectations, our study did not find a significant influence of PBD on OS after considering liver function parameters, including TB and serum albumin. What is surprising is that TB before intervention >150 μmol/L is an independent adverse prognostic factor both for DFS and OS in patients with obstructive jaundice.

Many previous studies have described PBD as an independent predictor for worse prognosis after pancreaticoduodenectomy for PDAC. In the study by Macias et al. (7), histologic grade (*P* = 0.019) and PBD (*P* = 0.016) were the sole independent variables predicting worse OS in patients with PDAC. In another study by Furukawa et al. (6), ERCP was associated with poor OS. Two other Japanese studies demonstrated that PTCD was an independent prognostic factor both for peritoneal recurrence and poor OS (8, 9). However, recent research based on the Surveillance, Epidemiology, and End Results (SEER) database found that ERCP before pancreaticoduodenectomy was not associated with long-term survival in patients >65 years old with pancreatic head cancer after using propensity score matching analysis (10).

Nevertheless, the previous studies, as mentioned above, may have selection bias and confounding bias. Selection of the patient population who received PBD to compare with the patient population who did not receive PBD was probably a major bias. A substantial proportion the patient population who did not receive PBD wasn't associated with obstructive jaundice. Not only did these patients not have a plan for PBD in any case but also the presence of jaundice was found to be a negative risk factor in patients with PDAC (11, 12). Thus, patients who did not receive PBD including those without obstructive jaundice, may lead to an apparent selection bias. Therefore, our study selected patients with obstructive jaundice as a specific population to eliminate the potential selection bias.

On the other hand, most of the previous studies did not include liver function parameters in their statistical analyses due to missing data or incomplete data, which may relate to confounding bias. Our study found that TB before intervention >150 μmol/L is an independent adverse prognostic factor in patients with obstructive jaundice with PDAC. In accordance with the present results, a high-quality randomized controlled trial (RCT) has demonstrated that high TB at randomization was found to be a worse prognostic factor for OS after pancreaticoduodenectomy in patients with PDAC, while PBD had no impact on OS (13). The patients with severely obstructive jaundice were more likely to have acute cholangitis and poor nutrition status (18). Hence, high TB could eventually lead to more episodes of PBD before surgery in clinical practice, aiming to reduce post-operative complications. Our data analyses also corroborate this conclusion, the PBD group had a higher rate of TB before intervention >150 μmol/L compared with the no PBD group (87.6 vs. 51.6%, *P* < 0.001) (Table 2).

After we adjusted for potential confounding factors by including liver function parameters in model 2 of the multivariate analyses for OS, PBD was no longer significant compared with model 1 (Table 3). Additionally, TB before intervention not only was associated with OS but also was associated with DFS. This result may be explained by the fact that patients with severely obstructive jaundice (TB before intervention >150 μmol/L) was the root cause of poor survival, instead of the PBD procedure. These results are in accordance with several previous studies indicating that high TB negatively influences long-term survival in patients with PDAC or periampullary carcinomas (18–20). This result may be explained by the fact that tumor aggressiveness in patients with high TB levels was stronger than patients with low TB levels. Another possible explanation for this is that severe obstructive jaundice may relate to immunosuppression, causing micrometastasis formation of tumors before intervention (21–23). Further experimental studies are needed to identify the relationship between the TB level and nature of pancreatic cancer.

Elderly patients, high NLR, surgery with venous resection and positive resection margins were found to be associated with both OS and DFS after resection. The elapsed time between PBD and surgery did not influence OS or DFS. These results are consistent with those of previous studies (13, 24–27). On the other hand, CA19-9, which is regarded as the most important tumor marker in patients with PDAC, was not included in our study. Although several studies concluded that elevated CA19-9 levels could predict early recurrence and OS after pancreatic resection in patients with PDAC (28–30), a high degree of collinearity is likely present between CA19-9 and TB before intervention based on the past research (31).

There are also several limitations to our study. First, as in any retrospective analysis, there may have been selection bias as well as incomplete information due to the retrospective design. A prospective validation study to confirm the possible risks of TB before intervention is necessary. Second, this study included patients with stage T4 due to the study design. Nevertheless, adjuvant chemotherapy is recommended for all patients with borderline resectable PDAC according to the recent NCCN guideline (4). Third, with the development of the endoscopic ultrasound (EUS) technique, EUS can be used as a preferable alternative drainage technique. However, we didn't perform EUS-guided biliary drainage before 2019, although EUS was widely used in our center.

In conclusion, PBD did not influence long-term OS or DFS in patients with obstructive jaundice with resectable PDAC who underwent pancreaticoduodenectomy, as compared with surgery alone. However, TB before intervention >150 μmol/L is an independent adverse prognostic factor both for DFS and OS in patients with obstructive jaundice. PBD remains the treatment of choice if there is a reasonable indication, considering the risks of the procedure and surgery.

## Data Availability Statement

The raw data supporting the conclusions of this article will be made available by the authors, without undue reservation.

## Ethics Statement

The studies involving human participants were reviewed and approved by Ruijin Hospital Ethics Committee Affiliated to Shanghai JiaoTong University School of Medicine, Shanghai, China. The patients/participants provided their written informed consent to participate in this study.

## Author Contributions

ZS, JZ, and HC: study conception, design, and drafting of the manuscript. ZS, HC, WW, WX, YZ, XL, SZ, ZX, and XD: acquisition of data. ZS, JZ, and YW: analysis and interpretation of data. JW, YW, and BS: critical revision. All authors contributed to the article and approved the submitted version.

## Conflict of Interest

The authors declare that the research was conducted in the absence of any commercial or financial relationships that could be construed as a potential conflict of interest.

## Supplementary Material

Supplementary Figure 1 Utilizing X-tile analysis to determine the optimal cut-off level of age. **(A)** The graph shows that the optimal cut-off point has been determined by X-tile software. **(B)** Histogram and **(C)** Kaplan-Meier analysis were conducted using the optimal cut-off value.

Supplementary Figure 2 Utilizing X-tile analysis to determine the optimal cut-off level of the total serum bilirubin (TB) before surgery. **(A)** The graph shows that the optimal cut-off point has been determined by X-tile software. **(B)** Histogram and **(C)** Kaplan-Meier analysis were conducted using the optimal cut-off value.
