# Supplementary figures and images for: Does Pre-operative Biliary Drainage Influence Long-Term Survival in Patients With Obstructive Jaundice With Resectable Pancreatic Head Cancer?
Source: Front Oncol. 2020 Sep 16;10:575316. doi: 10.3389/fonc.2020.575316 (PMC7525216; doi:10.3389/fonc.2020.575316)

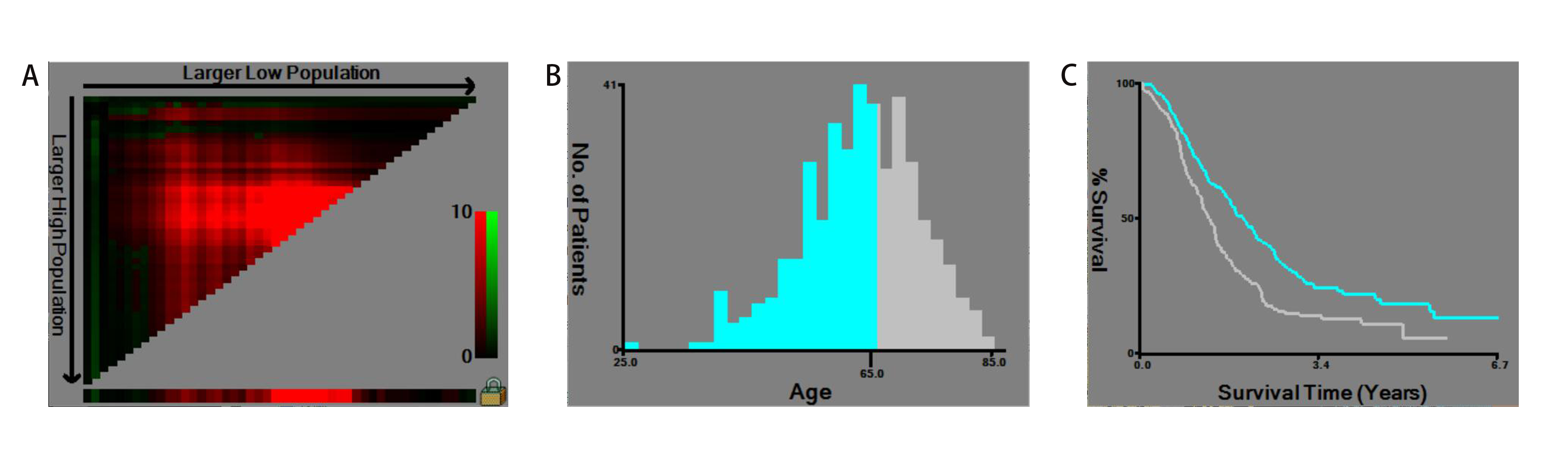

Supplement: Supplementary Figure 1 — Utilizing X-tile analysis to determine the optimal cut-off level of age. (A) The graph shows that the optimal cut-off point has been determined by X-tile software. (B) Histogram and (C) Kaplan-Meier analysis were conducted using the optimal cut-off value. [file Image_1.tif]

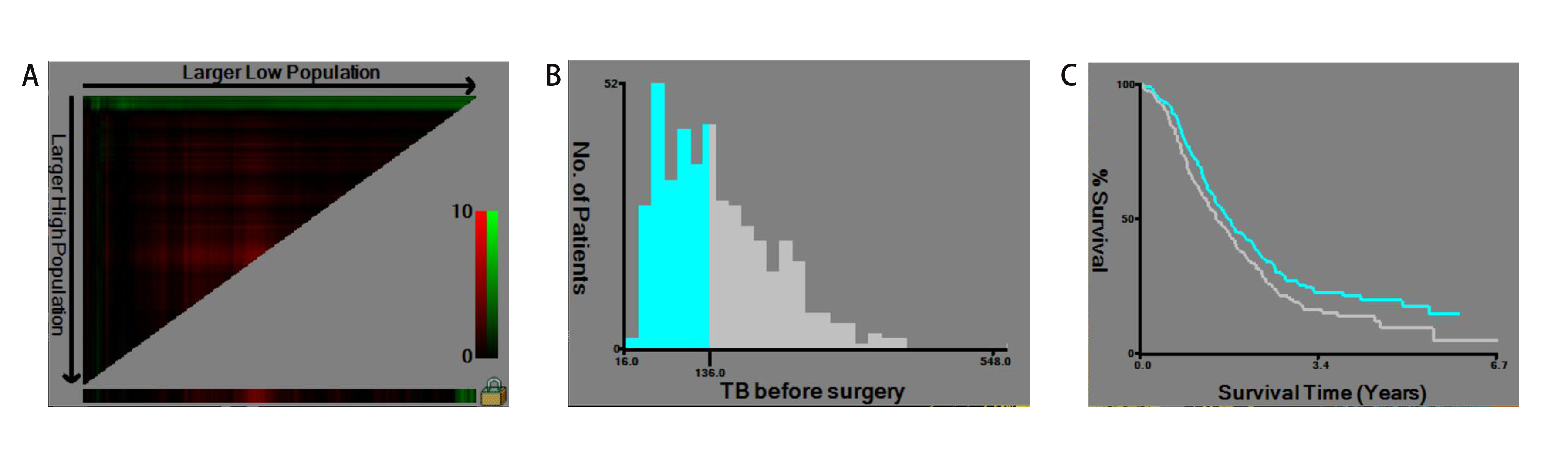

Supplement: Supplementary Figure 2 — Utilizing X-tile analysis to determine the optimal cut-off level of the total serum bilirubin (TB) before surgery. (A) The graph shows that the optimal cut-off point has been determined by X-tile software. (B) Histogram and (C) Kaplan-Meier analysis were conducted using the optimal cut-off value. [file Image_2.tif]
